# High-efficiency ^90^Sr radio-photovoltaic cells based on waveguide light concentration structure

**DOI:** 10.1038/s41377-025-01875-1

**Published:** 2025-06-16

**Authors:** Tongxin Jiang, Sijie Li, Wenlong Yao, Lu Han, Lei Zhang, Xue Li, Lifeng Zhang, Xian Tang, Xin Li, Haisheng San

**Affiliations:** 1 Pen-Tung Sah Institute of Micro-Nano Science and Technology, Xiamen University, Xiamen, China; 2 China Institute of Atomic Energy, Beijing, China

**Keywords:** Solar energy and photovoltaic technology, Photonic devices

## Abstract

Radio-photovoltaic cells (RPVCs) are able to offer high reliability and extended operational lifetimes, making them ideal for harsh-environment applications. However, the two-stage energy conversion process inherently limits energy conversion efficiency (ECE). This study presents a novel RPVC design based on a waveguide light concentration (WLC) scheme, employing multilayer-stacked GAGG:Ce scintillation waveguides alternately loaded with ^90^Sr radioisotope sources. Electron beam irradiation tests revealed highly efficient radioluminescence (RL) emission from the edge surfaces of GAGG:Ce waveguide at electron energies exceeding 60 keV. A RPVC prototype incorporating 1.43 Ci of ⁹⁰Sr achieved a maximum output power (*P*_max_) of 48.9 μW, with an unprecedented ECE of 2.96%—the highest reported value for radioisotope-powered RPVCs to date. Furthermore, a multi-module integrated RPVC prototype demonstrated a *P*_max_ of 3.17 mW, with a short circuit current of 2.23 mA and an open circuit voltage of 2.14 V. Remarkably, the device exhibited only 13.8% RL performance degradation after a 50-year equivalent electron beam irradiation (total fluence: 5.625 × 10^18^ e/cm^2^), confirming exceptional radiation hardness. These findings demonstrate that the WLC-based RPVCs achieve both high power output and exceptional long-term stability, representing a substantial advancement for facilitating nuclear battery applications.

## Abstract (web summary)

A novel radio-photovoltaic cell was developed using a waveguide light concentration design, featuring multilayer-stacked GAGG:Ce scintillation waveguides interleaved with ⁹⁰Sr sources. The devices achieved a total energy conversion efficiency of 2.96% and demonstrated only 13.8% radioluminescence degradation after 50-year equivalent electron beam irradiation. The study results represent a significant advancement for facilitating nuclear battery applications.

## Graphical abstract

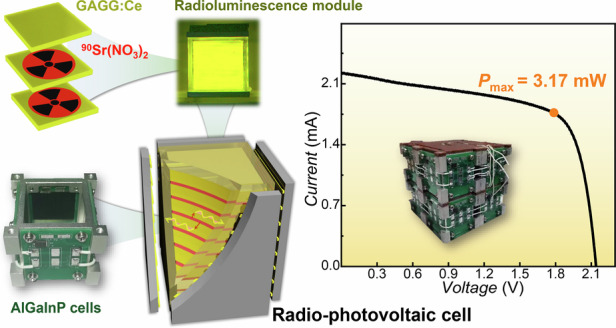

## Introduction

Nuclear batteries offer unique advantages for specialized applications in extreme environments, including space exploration, deep-sea operations, and polar/desert expeditions, garnering significant scientific and engineering interest^1–4^. Conventional power sources (e.g., chemical batteries, fuel cells, and photovoltaic cells) fail to meet the stringent operational demands of harsh environments, including long-term durability, maintenance-free operation, and continuous self-sustaining capabilities^5–8^. Nuclear batteries are categorized into various types based on their distinct energy conversion mechanisms^9,10^. Among them, radioisotope thermoelectric generators (RTGs), which directly convert radioactive-decay heat into electricity, have been applied in space exploration since the 1970s^11,12^. However, their main characteristics (e.g., large size and weight, super-high cost, and limited availability of ^238^Pu source) make them difficult to use extensively in commercial and civilian fields. Radiovoltaic cells (RCs) can operate by directly converting decay energy into electrical energy and are composed of radiation sources and semiconductor transducers. However, their service lifetimes are constrained by radiation-induced degradation of semiconductor materials^13,14^. Radio-photovoltaic cells (RPVCs) are a typical example of indirect conversion of radiation energy to electrical energy^1^. In these cells, radioluminescence (RL) materials like scintillators and phosphors collect decay particles to produce luminescence, which is then further converted to electricity through a PV device^15^. Owing to the excellent radiation resistance of luminous materials, RPVCs offer significant advantages in mitigating radiation damage and protecting the sensitive PV cell from radiation^16–18^. However, the two-stage indirect conversion process faces a fundamental limitation: significantly lower energy conversion efficiency (ECE) compared to direct single-stage conversion. To improve the ECE of RPVCs, it is necessary to optimize individual component performance and coordinate their synergistic operation to maximize overall efficiency.

The PV cells serving as the photoelectric conversion component in RPVCs are required to match their spectral response with the radioluminescence (RL) and maintain a stable response to low-intensity light^19–21^. Recently, PV cells based on III-V compounds such as GaP, GaAs, AlGaAs, and AlGaInP have been increasingly used in RPVCs to enhance the conversion of light energy into electricity^22^. However, these PV cells have reached the limit for the efficient enhancement of RPVCs. Increasing research is focusing on developing novel luminous materials to improve RL performance, including nanosphere-coated phosphor layers^23^, inorganic perovskite quantum dots^24,25^, and scintillation crystals (e.g., GAGG:Ce, CsI: Tl, YAG:Ce)^26–28^. Furthermore, the coupling schemes of radiation sources and scintillators have been subjected to extensive investigation, including tritium gas-filled glass tubes coated with phosphor powder^29^, a mixture of ^63^NiCl_2_ solution and powdered phosphor^30^, and aerogel-phosphor composites saturated with tritium^31^. In these schemes, large and thin phosphors are required to enhance luminance and overcome the issue of light self-absorption within the phosphor materials. This necessitates an extended PV area to collect the emitted light flux, which is not helpful for the miniaturization and cost reduction of RPVCs. Waveguide light concentration (WLC) structure is considered promising scheme for addressing the above-mentioned issues^32^. Multilayer-stacked scintillation waveguides (SWs) with in-situ integrated radioisotopes between layers generate concentrated edge-emitted light, increasing photon flux density on PV cells to enhance photoelectric conversion efficiency. This design is not only beneficial for the miniaturization of RPVCs but also for the protection of PV cells from ionizing radiation. WLC-based structures require efficient scintillators with high transparency in their self-light. Although alkali halides (e.g., CsI, SrI_2_:Eu) and ZnS:Cu are efficient in RL performance, they also have shortcomings such as light self-absorption^33^ or high hygroscopicity^34^. Oxide scintillation crystals GAGG:Ce (Gd_3_Al_2_Ga_3_O_12_:Ce) with high photon yield, good radiation stability, and waveguide properties are ideal waveguiding materials for the PRVC applications^35–37^. However, so far, there have been few reports on their practical application in RPVCs.

The radiation source is another critical factor influencing the performance of RPVCs. Its parameters are determined by decay energy, specific activity, half-life, availability, and cost. Since the waveguide thickness must be comparable to the penetration depth of decay particles, the use of low-energy radiation sources (e.g., tritium, ^63^Ni, and ^14^C) requires micrometer-scale layers^38^. This will lead to some challenges in the fabrication and assembly of waveguide components. Therefore, high-energy radiation sources (e.g., ^137^Cs, ^90^Sr, and ^241^Am) are recommended for use, not only to ensure the operability of thick-layer waveguides but also to meet the commercial requirements for high power output^39^. However, the radiation hardness of SWs exposed to high-energy radioisotopes requires systematic evaluation.

In this work, a WLC-based RPVC was designed and fabricated using multilayer-stacked GAGG:Ce SWs interleaved with the ^90^Sr radiation sources. The structural parameters of the SWs were optimized using Monte Carlo (MC) simulations, and the RL performance and radiation stability of the scintillators were investigated using electron beam (EB) irradiation across a wide range of energies and beam fluxes. The ^90^Sr-powered RPVC prototype based on AlGaInP-based photovoltaics demonstrated a significant enhancement in both ECE and output power, validating the efficient WLC scheme.

## Results

**Fig. 1:**
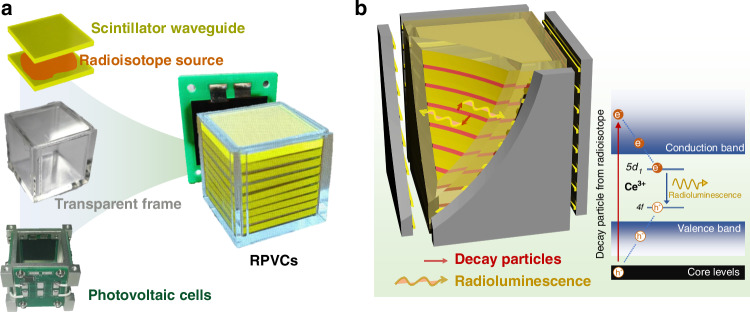
The structure and conversion principle of the WLC-based RPVC. **a** Integrated component photos of the WLC-based RPVC and **b** schematic 3-D structural diagram of the RL module based on multilayer-stacked GAGG:Ce SWs interleaved with ^90^Sr films. Inset is a schematic illustration of RL generation and transmission in the Ce^3+^-doped scintillators

Figure 1a shows a schematic diagram of a WLC-based RPVC, which consists of a multi-layer stacked SW module, a quartz container, and four AlGaInP-based PV cells. Square SW plates are alternately loaded with the radioisotope films, which are encased in a radiation-resistant quartz container to form an RL module. Four AlGaInP-based PV cells are attached to the four faces of the glass container, aligning with the emitting edges of the waveguide plates to collect the concentrated light, as illustrated in Fig. 1b. The PV cells are specifically designed to well-match their spectral response with the emission spectrum of the scintillator. The pure β-emitting ^90^Sr radioisotope was used as the radiation source due to its relatively low cost, long half-life (*T*_1/2_ = 28.9 years), high decay energy, and widespread availability. The high-energy particles penetrate the SWs to generate RL photons, which are guided along the transverse direction to the four edges of the waveguide plates and then concentrated into a slender beam of light. The thin radioisotope films are sandwiched between two scintillation plates, which not only reduce the self-absorption of β particles within the radiation source but also allow for the complete absorption of decay particles by the scintillators from all angles. The GAGG:Ce crystal (Gd_3_Al_2_Ga_3_O_12_:Ce) was used for the fabrication of SW owing to its high scintillation yield of 55,000 ph/MeV and high refractive index (*n* = 1.96). The doping of Ce^3+^ ions allow its excited energy level of 4 f → 5 d to be lower than the wide bandgap of GAGG:Ce, thereby resulting in the generation of high-yield RL photons and a high transmittance to its own emission photons. With its high refractive index, the GAGG:Ce scintillator exhibits near-total internal reflection at its polished surfaces, making it an ideal candidate for low-loss waveguide applications.

**Fig. 2:**
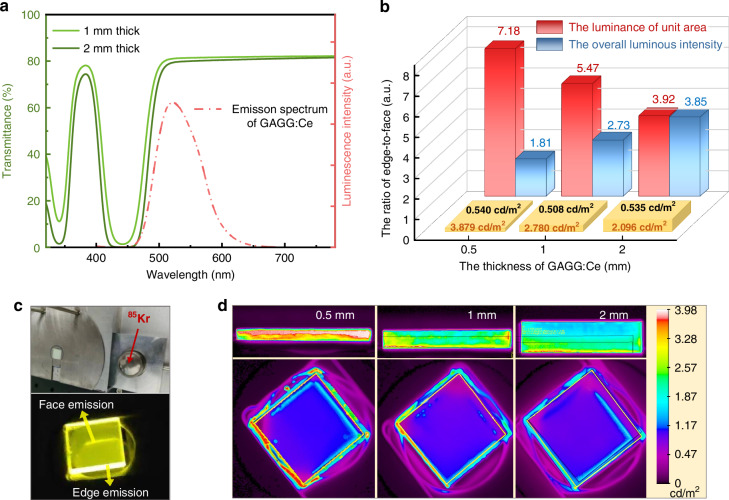
Enhanced emission verification of the WLC structure. **a** Transmittance and PL spectra of GAGG:Ce scintillator. **b** Edge-to-face RL intensity ratios. **c** ^85^Kr radiation platform and GAGG:Ce SW under ^85^Kr irradiation. **d** Comparative RL intensity profiles at polished surfaces and roughness edges for 0.5 mm, 1 mm, 2 mm thick GAGG:Ce SWs

The transmittance and emission spectra of GAGG:Ce scintillators are shown in Fig. 2a. Two absorption bands are observed with peaks at 440 nm (2.65 eV) and 340 nm (3.65 eV), corresponding to the spin-and-parity-allowed electronic transitions of 4 f → 5d_1_ and 4 f → 5d_2_ of Ce^3+^, respectively. The transmittance spectra show a transmittance of 80% for wavelengths greater than 510 nm, covering the main portion of the photoluminescence (PL) spectrum of the GAGG:Ce scintillator in the range of 450 nm to 650 nm. The thickness variation of SWs has a slight effect on transmittance, which can be negligible.

In the WLC structure, both main faces of the SWs were polished to enhance the total internal reflection, and the four edges were roughened to enhance edge emission (Supplementary Fig. S1). It is demonstrated that a rough surface can significantly enhance the light emission in comparison with the polished and antireflective surfaces, as shown in Supplementary Table S1. To compare the light emission intensity from the main face and edge of GAGG:Ce waveguides with different thicknesses (e.g., 0.5 mm, 1 mm, and 2 mm), the RL from both surfaces was measured using a ^85^Kr radiation platform (Fig. 2c), and their edge-to-face luminance intensity ratios are shown in Fig. 2b. According to the β-energy spectrum data of ^85^Kr (Supplementary Table S2), the maximum penetration depth of β-particles emitted from the ^85^Kr in the GAGG:Ce scintillator was estimated to be 300 μm based on MC simulations (MCNP5, Monte Carlo N Particle Transport Code), as shown in Supplementary Fig. S2. Since the thickness of the waveguides is significantly greater than the penetration depth of the β-particles, the RL measured from the main faces of waveguides with varying thicknesses remains nearly the same, with an average of 0.528 ± 0.02 cd/m^2^. In contrast, the RL of edge emissions is significantly higher than those of face emissions. As shown in Fig. 2c, the maximum luminance of edge emission reaches 3.879 cd/m² in a 0.5 mm thick waveguide, making a maximum edge-to-face luminance ratio of 7.18, in comparison with ratios of 5.45 for a 1 mm one and 3.92 for a 2 mm one, respectively. These results directly verify the light-concentration effect in SWs. Figure 2d exhibits the comparative RL intensity profiles at polished surfaces and roughness edges for the GAGG:Ce SWs with varying thicknesses. A reduction in edge emission luminance was observed with increasing waveguide thickness. This can be explained by the fact that thick waveguides suffer from luminance dispersion across larger edge areas, whereas thin waveguides can enhance luminance intensity through photon spatial compression. While thinner waveguides exhibit higher internal reflection losses, the increased waveguide thickness enhances total luminous intensity by expanding the effective edge emission area. This suggests that a thicker waveguide is capable of achieving higher RL efficiency under identical excitation conditions. Considering the cost-efficiency requirement for practical applications, the optimum thickness of the waveguide was determined to be 2 mm.

**Fig. 3:**
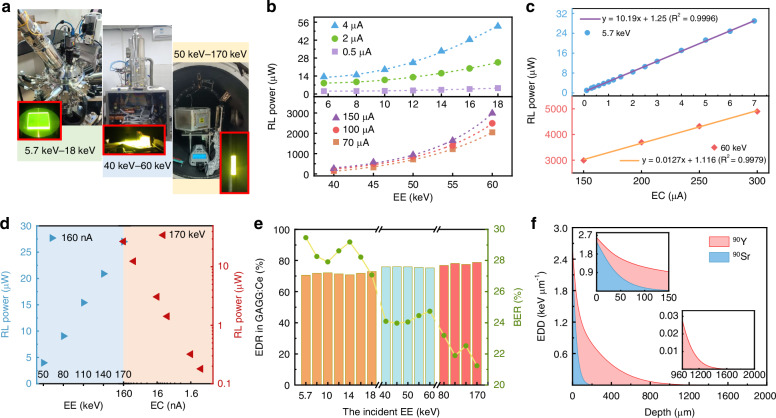
The rule of EE and EC on the optical performance of GAGG:Ce SWs. **a** Photos of EB generators with different EE levels. **b** Dependences of RL powers on the low- and medium-energy EB with various ECs. **c** Dependences of RL powers on the ECs in the EEs of 5.7 keV and 60 keV. **d** Dependences of RL powers on the high-energy EB with various ECs and EEs. **e** EE-dependent EDR and BER in GAGG:Ce. **f** Depth-dependent EDD in GAGG:Ce under ^90^Sr/^90^Y radiation

EB irradiation was used to simulate the β emission of radiation sources, allowing for precise control of the electron energy (EE) and electron current (EC) for flexible and targeted experimental research. Three different EB generators were used to generate EBs with different EE levels (Fig. 3a), corresponding to a low-energy EB with an EE range from 5.7 keV to 18 keV, a medium-energy EB with an EE range from 40 keV to 60 keV, and a high-energy EB with an EE range from 50 keV to 170 keV, respectively. A light power meter was positioned adjacent to the emission edge of the SW within the vacuum chamber to continuously in-situ monitor the RL power. Figure 3a shows the effects of low- and middle-energy EBs with various ECs on the RL of edge emission in the 2-mm-thick GAGG:Ce SW, respectively. It can be observed that the RL power increases with an increase in the EE and EC, and the nonlinear increase in light power implies that the RE can be increased by enhancing the incident EE. Low-energy electrons deposit energy predominantly near the surface of scintillators, resulting in a localized photon emission zone. With the increase in EE, the spatial distribution of luminescent zone expands correspondingly, leading to enhanced photon emission efficiency in GAGG:Ce scintillators. Furthermore, the influence of the EC on RL power exhibits a linear increase trend, which can be further verified by Fig. 3b. With the use of a high-energy EB, the increases in RL power are linearly dependent on the increase in both the EE and EC, as shown in Fig. 3c. This means that the RL efficiency remains relatively stable for high-energy EB radiation. This could be attributed to the multiple excitation effect, which generates amounts of secondary electrons through the continuous collision of high-energy electrons with the atomic structure of the scintillator. These secondary electrons further excite more 5d_1_ → 4 f transitions of Ce^3+^ in the scintillator, thereby resulting in enhanced light-emission and a stable RL efficiency. The three EB devices, along with their corresponding test images, are depicted in Fig. 3d.

MCNP simulations were conducted to determine the effective energy deposition of electrons in the GAGG:Ce scintillator and the energy backscattering loss of incident electrons. As shown in Fig. 3e, with an increase in the incident EE, the backscattering electron rate (BER) decreases, thus leading to an increase in the energy deposition rate (EDR). Low-energy electrons can increase the BER by 10% compared to high-energy electrons. This suggests that high-energy β-emitting sources are more preferable for the GAGG:Ce scintillator than low-energy ones. In this work, the ^90^Sr radioisotope was used as the radiation source owing to its high average energy of 195.8 keV. With β-decay, ^90^Sr will decay into ^90^Y, which subsequently undergoes β-decay to produce stable ^90^Zr. Owing to the much shorter half-life of ^90^Y (64 h) compared to ^90^Sr, ^90^Sr and ^90^Y coexist with each ^90^Sr β-decay^40^. Therefore, the β-particles of ^90^Sr/^90^Y have an average energy of ∼ 0.3 MeV and a maximum energy of ∼2.2 MeV. Additionally, ^90^Y will emit a gamma (γ) ray when undergoing β-decay. Figure 3f shows the depth-dependent energy deposition density (EDD) in GAGG:Ce under ^90^Sr/^90^Y irradiation, which was calculated through MCNP simulation. A total of 99% of the β-decay energy is deposited within a depth of 960 μm, and the remaining energy can be deposited completely within a 2 mm depth. The EDD for both ^90^Sr and ^90^Y are also presented in the insets of Fig. 3f, corresponding to their respective energy spectra (Supplementary Fig. S3 and Tables S3, S4). It can be observed that the β-decay energy of ^90^Y is crucial for determining the optimum depth of the SW.

**Fig. 4:**
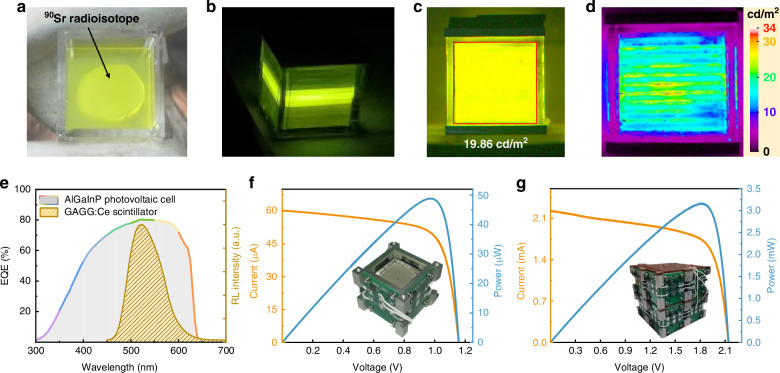
Prototype preparation and performance testing of the WLC-based RPVC. **a** Top-view photo of gel-based ^90^Sr(NO_3_)_2_ solution adhered to the surface of SW. **b** Photo of RL module loaded with two-layer ^90^Sr film. **c** Photo of RL module loaded with multilayer ^90^Sr film. **d** RL intensity profile of RL module. **e** EQE curve of AlGaInP-based PV cells and PL spectrum of GAGG:Ce scintillator. *I-V* and *P-V* curves for RPVC prototypes with (**f**) single module and (**g**) 64 modules

The WLC-based RPVC prototype was assembled using a remotely operated manipulator in a closed glove box for radiation isolation and protection. The GAGG:Ce scintillation crystal was cut into 25 mm × 25 mm × 2 mm plates. A gel-based ^90^SrNO_3_ solution was coated on the surface of the waveguide plates. After heating and drying, a radioisotope film was adhered to the surface of each waveguide plate (Fig. 4a). Figure 4b shows an RL module with bright RL in the middle region of the multilayer-stacked GAGG:Ce waveguides. The RL was measured up to 0.6225 cd/m^2^, corresponding to a ^90^Sr activity of 43.5 mCi in two-layers of ^90^Sr films. Figure 4c demonstrates an RL module interleaved with ^90^Sr films in a ten-layer stacked SW, with a total activity of 1.43 Ci. The average RL across the entire emission surface reached 19.86 cd/m^2^. Figure 4d shows the RL intensity profile across the entire emission surface. The central region of the module exhibits higher luminance than the surrounding region, with the maximum luminance reaching ∼34 cd/m^2^. It is suggested that there is a small portion of light that leaks out of the waveguides and enters into the adjacent waveguides, thereby resulting in a overlay enhancement of emission light in the central region. Additionally, the radiation sources were not fully covered on the entire surface of the waveguides, as shown in Fig. 4a, which is responsible for the relatively weak light emission in the surrounding region. The external quantum efficiency (EQE) curve of the AlGaInP-based PV cells was also measured, as shown in Fig. 4e. It can be observed that the EQE curve well-matches the PL spectrum of the GAGG:Ce scintillator, indicating a successful design and fabrication of AlGaInP-based PV cells for efficient conversion of light energy to electricity. Figure 4f shows the current-voltage (*I-V*) and power-voltage (*P-V*) curves of the WLC-based RPVC prototype. The short-circuit current (*I*_sc_) and open-circuit voltage (*V*_oc_) were measured to be 60.3 μA and 1.16 V, respectively, and the maximum output power (*P*_max_) reached 48.9 μW. The overall ECE was calculated to be 2.96% (Calculation method in Supplementary Information), and it is a record ECE value among the reported RPVCs (Supplementary Table S5). A multi-module integrated RPVC prototype was assembled by integrating and cascading connection of 64 modules, which were encapsulated within a plumbum container during experiments, ensuring no leakage of γ-ray. The *I-V* and *P-V* curves were measured as shown in Fig. 4g. The *P*_max_ reached 3.17 mW with an *I*_sc_ of 2.23 mA and a *V*_oc_ of 2.14 V.

**Fig. 5:**
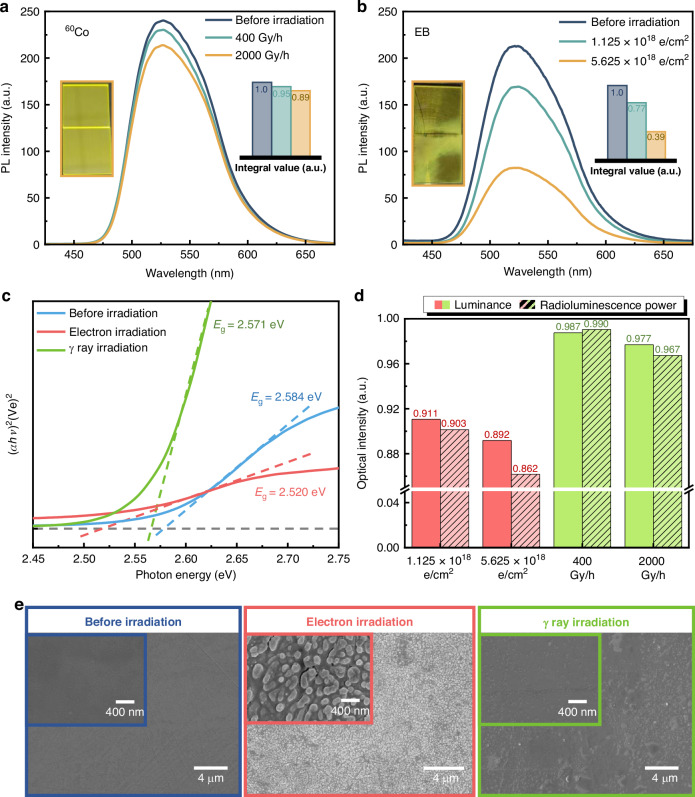
Irradiation damage and performance degradation of GAGG:Ce SWs. **a** PL spectra of GAGG:Ce scintillators before and after *γ*-radiations with 400 Gy/h and 2000 Gy/h. **b** PL spectra of GAGG:Ce SWs before and after EB-irradiations with 1.125 × 10^18^ e/cm^2^ and 5.625 × 10^18^ e/cm^2^. **c** Bandgap estimations of GAGG:Ce scintillators before and after EB- and γ-irradiations. **d** Effects of EB- and γ-irradiations on RL performance. **e** SEM images of GAGG:Ce surfaces before and after EB- and γ-irradiations

Owing to the direct contact with the ^90^Sr radioisotope, the GAGG:Ce SWs are continuously exposed to high-energy β- and γ-irradiations over prolonged periods. These decay energies excite the scintillator while potentially causing radiation damage to it. It is necessary to investigate the radiation tolerance of GAGG:Ce SWs under β- and γ-irradiations. An EB (simulated β-radiation source) source and a ^60^Co γ-irradiation source were used to irradiate GAGG:Ce SWs, respectively. For the γ-irradiation tests, two different dose rates of 400 Gy/h and 2000 Gy/h were used by controlling the irradiation duration to deliver a total dose of 2.89 × 10^5 ^Gy, respectively. For the EB*-*irradiation tests, two 60 keV electron fluxes of 6.25 × 10^14^ e/cm^2^·s and 3.125 × 10^15^ e/cm^2^·s were applied for 30 minutes, resulting in total fluences of 1.125×10^18^ e/cm^2^ and 5.625 × 10^18^ e/cm^2^, respectively. These fluences correspond to approximately the same amount of β emission from a radiation source of 100 mCi/cm^2^ over periods of 10 and 50 years, respectively. The RL performance and surface morphology of the GAGG:Ce SWs were analyzed before and after irradiation, as shown in Fig. 5. It was found that the intensities of the PL spectra decreased by 5% and 11% after γ-irradiations with 400 Gy/h and 2000 Gy/h, respectively, when compared with those before γ-irradiation (Fig. 5a). An increased dose rate results in proportionally enhanced degradation in RL performance at identical total irradiation doses. In contrast, EB-irradiation has a more significant impact on the intensity of PL spectra than γ-irradiation. As shown in Fig. 5b, EB-irradiations with fluences of 1.125 × 10^18^ e/cm^2^ and 5.625 × 10^18^ e/cm^2^ lead to reductions of 23% and 61% in the PL intensity, respectively. Distinctive differentiation features were prominently observed on the GAGG:Ce surface, as depicted in the inset of Fig. 5b, which are primarily responsible for the notable decrease in the PL spectra intensity. Measurements of the absorption spectra were used to provide insight into the effects of EB- and γ-irradiations on the optical properties. Figure 5c shows the bandgap values of GAGG:Ce scintillators before and after EB- and γ-irradiations, calculated from their main optical absorption edges. In comparison with the original bandgap value of 2.584 eV, the bandgap values after EB- and γ-irradiations were changed to 2.520 eV and 2.571 eV, respectively, suggesting that EB-irradiation has a greater impact on the bandgap than γ-irradiation.

Figure 5d shows the effects of EB- and γ-irradiations on RL edge-emission. With the same irradiation time, a higher electron flux will lead to greater degradation in both luminance and RL power. After EB-irradiation with a fluence of 5.625 × 10^18^ e/cm^2^, the maximum degradations in luminance and RL power reached 10.8% and 13.8%, respectively. In contrast, γ-irradiation with a dose rate of 2000 Gy/h exerts less impact on luminance and RL power than EB-irradiation, resulting in only 2.3% and 3.3% degradation, respectively. By observing SEM images of GAGG:Ce surfaces before and after EB- and γ-irradiations (see Fig. 5e), EB-irradiation induces substantial surface damage through the formation of microtrenches induced by electron etching. In contrast, the surface exposed to γ-irradiation is relatively smooth owing to the high penetrating ability of γ-rays. The formation of microtrenches increases surface roughness in waveguide surfaces, thereby leading to enhanced light leakage through these surfaces and reducing the edge emission efficiency.

## Discussion

In summary, a WLC-based RPVC was designed and fabricated using multilayer-stacked GAGG:Ce SWs interleaved with the ^90^Sr radiation sources, which demonstrated efficient radiation-to-electrical energy conversion and excellent radiation stability. Through the Monte Carlo simulation to optimize WLC structure, the light collection efficiency of RL model was enhanced, and thus the total ECE of RPVC was improved. The 2-mm-thick GAGG:Ce SW is well-suited for the radiation penetration depth of high-energy electrons emitted from ^90^Sr. The enhanced RL emission from the waveguide edges was verified using an β-irradiation from ^85^Kr source, showing an enhancement of 2.85 times in comparison with the RL emission from the main faces of SW. EB-irradiation tests revealed highly efficient RL emission from the waveguide edge at electron energies exceeding 60 keV. Remarkably, the GAGG:Ce SWs exhibited only 13.8% RL degradation after a 50-year equivalent EB-irradiation (total fluence of 5.625 × 10¹⁸ e/cm²), demonstrating exceptional radiation hardness. The AlGaInP-based PV cells used in the devices were well-matched to the GAGG:Ce emission spectrum. A WLC-based RPVC prototype loaded with 1.43 Ci of ^90^Sr achieved a *P*_max_ of 48.9 μW, with an ECE of 2.96%—the highest reported value for radioisotope-powered RPVCs. A 64-module prototype demonstrated a *P*_max_ of 3.17 mW with *I*_sc_ = 2.23 mA and *V*_oc_ = 2.14 V. Although large-scale production of RPVCs remains constrained by both challenges, namely mass production and cost reduction of ^90^Sr radioisotope, the current research results demonstrate a substantial advancement for facilitating nuclear battery applications.

## Materials and methods

### Materials and radiation sources

GAGG:Ce scintillators were available from the 26th Institute of China Electronics Technology Group Corporation (The density is 6.63 g/cm^3^); The ^90^Sr(NO_3_)_2_ solution and ^85^Kr gas were provided by the China Institute of Atomic Energy; AlGaInP-based PV cells was provided by Shanghai Institute of Space Power-Sources; Electron emitters with various electron energy ranges (e.g., 5.7–18 keV, 40–60 keV, and 50–170 keV) were used as simulated radiation sources, which were obtained from the China Institute of Atomic Energy, the Institute of Electrical Engineering (Chinese Academy of Sciences), and Harbin Institute of Technology, respectively.

### Characterizations

The transmittance and absorbance of the scintillators were measured using a UV/Vis/NIR spectrophotometer (Varian, Cary-5000). A light power meter (Ophir, PD300-UV) and an imaging colorimeter (Radiant Vision Systems, ProMetric I) were used to measure the luminescence performance of GAGG:Ce SWs. The emission spectrum of the scintillators was measured using a fluorescence spectrophotometer (HITACHI, F-7000). The electrical performance of the RPVCs was characterized using a digital source meter (Keithley, 2636). The morphological characteristics and elemental analysis were performed using scanning electron microscopy (Zeiss, SUPRA55 SAPPHIRE). The microscopic surface roughness was measured using an atomic force microscope (Cypher S, Asylum Research). The morphology of the frosted surfaces was analyzed using a profile-analyzing laser microscope (VK-X250K, Keyence) and a step profiler (Dektak-XT, Bruker).

## Supplementary information

Supplementary Information for High-efficiency <sup>90</sup>Sr radio-photovoltaic cells based on waveguide light concentration structure

## Data Availability

The data supporting this study’s findings are available from the corresponding author upon reasonable request.

## References

[1] Li, K. et al. Micronuclear battery based on a coalescent energy transducer. *Nature* **633**, 811–815, 10.1038/s41586-024-07933-9 (2024). 39294377 10.1038/s41586-024-07933-9

[2] Kumar, S. Atomic batteries: energy from radioactivity. *J. Nucl. Energy Sci. Power Gener. Technol.* **5**, 1, 10.4172/2325-9809.1000144 (2016).

[3] Prelas, M. A. et al. A review of nuclear batteries. *Prog. Nucl. Energy* **75**, 117–148, 10.1016/j.pnucene.2014.04.007 (2014).

[4] Olsen, L. C., Cabauy, P. & Elkind, B. J. Betavoltaic power sources. *Phys. Today* **65**, 35–38, 10.1063/PT.3.1820 (2012).

[5] Kim, H. S. et al. Multiple-year battery based on highly efficient and stable dual-site radioactive isotope dye-sensitized betavoltaic cell. *J. Power Sources* **606**, 234427, 10.1016/j.jpowsour.2024.234427 (2024).

[6] Terranova, M. L. Nuclear batteries: current context and near-term expectations. *Int. J. Energy Res.* **46**, 19368–19393, 10.1002/er.8539 (2022).

[7] Iwan, A., Pellowski, W. & Bogdanowicz, K. A. Conversion of radiophotoluminescence irradiation into electricity in photovoltaic cells. A review of theoretical considerations and practical solutions. *Energies* **14**, 6186, 10.3390/en14196186 (2021).

[8] Lange, R. G. & Carroll, W. P. Review of recent advances of radioisotope power systems. *Energy Convers. Manag.* **49**, 393–401, 10.1016/j.enconman.2007.10.028 (2008).

[9] Naseem, M. B. et al. Betavoltaic nuclear battery: a review of recent progress and challenges as an alternative energy source. *J. Phys. Chem. C.* **127**, 7565–7579, 10.1021/acs.jpcc.3c00684 (2023).

[10] Spencer, M. G. & Alam, T. High power direct energy conversion by nuclear batteries. *Appl. Phys. Rev.* **6**, 031305, 10.1063/1.5123163 (2019).

[11] Rowe, D. M. Applications of nuclear-powered thermoelectric generators in space. *Appl. Energy* **40**, 241–271, 10.1016/0306-2619(91)90020-X (1991).

[12] O’Brien, R. C. et al. Safe radioisotope thermoelectric generators and heat sources for space applications. *J. Nucl. Mater.* **377**, 506–521, 10.1016/j.jnucmat.2008.04.009 (2008).

[13] Eiting, C. J. et al. Demonstration of a radiation resistant, high efficiency SiC betavoltaic. *Appl. Phys. Lett.* **88**, 064101, 10.1063/1.2172411 (2006).

[14] Jiang, T. X. et al. Research on output power of radio-voltaic nuclear battery. 2020 Asia Energy and Electrical Engineering Symposium. 763–766, (IEEE, 2020). 10.1109/AEEES48850.2020.9121369 (2020).

[15] Jiang, T. X. et al. ^63^Ni-based radioluminescent isotope cells with enhanced photon transport interfaces. *J. Sci.: Adv. Mater. Devices* **8**, 100611, 10.1016/j.jsamd.2023.100611 (2023).

[16] Sychov, M. et al. Alpha indirect conversion radioisotope power source. *Appl. Radiat. Isotopes* **66**, 173–177, 10.1016/j.apradiso.2007.09.004 (2008). 10.1016/j.apradiso.2007.09.004 17977736

[17] Sharma, A. et al. Novel use of semiconductive conjugated polymer with optimized scintillator for betavoltaic applications. ASME 2015 International Mechanical Engineering Congress and Exposition. 50739, (ASME, 2015). 10.1115/IMECE2015-50739 (2015).

[18] Li, X. M. et al. Mn^2+^ induced significant improvement and robust stability of radioluminescence in Cs_3_Cu_2_I_5_ for high-performance nuclear battery. *Nat. Commun.* **12**, 3879, 10.1038/s41467-021-24185-7 (2021). 34162878 10.1038/s41467-021-24185-7 PMC8222237

[19] Jiang, T. X. et al. In-depth analysis of the internal energy conversion of nuclear batteries and radiation degradation of key materials. *Energy Technol.* **8**, 2000667, 10.1002/ente.202000667 (2020).

[20] Wu, Y. S. et al. Research on X-ray-based energy conversion technology and assessment of application prospect. *Sustain. Energy Technol. Assess.* **60**, 103552, 10.1016/j.seta.2023.103552 (2023).

[21] Russo, J., Ray, W. I. I. & Litz, M. S. Low light illumination study on commercially available homojunction photovoltaic cells. *Appl. Energy* **191**, 10–21, 10.1016/j.apenergy.2017.01.029 (2017).

[22] Zhao, C. et al. Tenfold efficiency improvement of x-ray radioluminescent batteries basing on GAGG: Ce single crystal scintillators. *Appl. Phys. Lett.* **119**, 223901, 10.1063/5.0073048 (2021).

[23] He, Y. H. et al. Enhanced radioluminescence and improved radioluminescent nuclear battery output performance more than 50% with SiO_2_ nanosphere coating. *J. Lumin.* **255**, 119600, 10.1016/j.jlumin.2022.119600 (2023).

[24] Yang, D. D. et al. Armor-like passivated CsPbBr_3_ quantum dots: boosted stability with hand-in-hand ligands and enhanced performance of nuclear batteries. *J. Mater. Chem. A* **9**, 8772–8781, 10.1039/d0ta12365j (2021).

[25] Gao, R. L. et al. High efficiency formamidinium-cesium perovskite-based radio-photovoltaic cells. *Energy Environ. Mater.* **7**, e12513, 10.1002/eem2.12513 (2024).

[26] Karpyuk, P. et al. The saturation of the response to an electron beam of Ce-and Tb-doped GYAGG phosphors for indirect β-voltaics. *Appl. Sci.* **13**, 3323, 10.3390/app13053323 (2023).

[27] Korzhik, M. et al. Towards effective indirect radioisotope energy converters with bright and radiation hard scintillators of (Gd,Y)_3_Al_2_Ga_3_O_12_ family. *Nucl. Eng. Technol.* **54**, 2579–2585, 10.1016/j.net.2022.02.007 (2022).

[28] Zhao, C. et al. X-ray radioluminescent battery with near milliwatt output power using CsI: Tl single crystal scintillator. *Appl. Phys. Lett.* **121**, 123906, 10.1063/5.0109011 (2022).

[29] Walton, R. et al. Radioisotopic battery and capacitor system for powering wireless sensor networks. *Sens. Actuators A Phys.* **203**, 405–412, 10.1016/j.sna.2013.09.010 (2013).

[30] Russo, J. et al. A radioluminescent nuclear battery using volumetric configuration: ^63^Ni solution/ZnS: Cu, Al/InGaP. *Appl. Radiat. Isotopes* **130**, 66–74, 10.1016/j.apradiso.2017.09.018 (2017). 10.1016/j.apradiso.2017.09.018 28942331

[31] Phatangare, A. B. et al. Novel nuclear batteries based on radioluminescence. *Energy Technol.* **10**, 2200285, 10.1002/ente.202200285 (2022).

[32] Bower, K. E. et al. Polymers, phosphors, and voltaics for radioisotope microbatteries. (CRC Press, 2002).

[33] Jiang, T. X. et al. Comparison and study of the preparation methods for phosphor layer in nuclear battery. *Int. J. Energy Res.* **45**, 11712–11720, 10.1002/er.5526 (2021).

[34] Xie, Y. G. et al. Influence of air exposure on CsI photocathodes. *Nucl. Instrum. Methods Phys. Res. Sect. A Accel. Spectrometers Detect. Assoc. Equip.* **689**, 79–86, 10.1016/j.nima.2012.06.023 (2012).

[35] Kamada, K. et al. 2 inch diameter single crystal growth and scintillation properties of Ce: Gd_3_Al_2_Ga_3_O_12_. *J. Cryst. Growth* **352**, 88–90, 10.1016/j.jcrysgro.2011.11.085 (2012).

[36] Yoneyama, M. et al. Evaluation of GAGG: Ce scintillators for future space applications. *J. Instrum.* **13**, P02023, 10.1088/1748-0221/13/02/P02023 (2018).

[37] Jarrell, J. T. et al. Radiation hardness of polycrystalline ceramic scintillators for radioisotope batteries. Hard X-Ray, Gamma-Ray, and Neutron Detector Physics XXIV. (SPIE, 2022) 10.1117/12.2635787 (2022).

[38] Moayedi, H. et al. Optimization of beta radioluminescent batteries with different radioisotopes: a theoretical study. *Nucl. Sci. Eng.* **195**, 614–625, 10.1080/00295639.2020.1848199 (2021).

[39] Lei, Y. S. et al. Demonstration and aging test of a radiation resistant strontium-90 betavoltaic mechanism. *Appl. Phys. Lett.* **116**, 153901, 10.1063/1.5140780 (2020).

[40] Dixon, J. et al. Evaluation of a silicon ^90^Sr betavoltaic power source. *Sci. Rep.* **6**, 38182, 10.1038/srep38182 (2016). 27905521 10.1038/srep38182 PMC5131278

