# Supplementary material for: High-efficiency 90Sr radio-photovoltaic cells based on waveguide light concentration structure
Source: Light Sci Appl. 2025 Jun 16;14:214. doi: 10.1038/s41377-025-01875-1 (PMC12170895; doi:10.1038/s41377-025-01875-1)
Supplement: Supplementary file 1 — Supplementary Information for High-efficiency 90Sr radio-photovoltaic cells based on waveguide light concentration structure [file 41377_2025_1875_MOESM1_ESM.docx]

Supplementary Information for

High-efficiency ^90^Sr radio-photovoltaic cells based on waveguide light concentration structure

Tongxin Jiang^a, 1^, Sijie Li^b, 1^, Wenlong Yao^a^, Lu Han^b^, Lei Zhang^b^, Xue Li^b,^ *, Lifeng Zhang^b^, Xian Tang^b^, Xin Li^b,^ *, Haisheng San^a,^ *

^a^ Pen-Tung Sah Institute of Micro-Nano Science and Technology, Xiamen University, Xiamen 361005, China

^b^ China Institute of Atomic Energy, Beijing 102413, China

^*^ Corresponding authors. E-mail addresses: sanhs@xmu.edu.cn (Haisheng San); lixin_0128@sina.com (Xin Li); lixue@ciae.ac.cn (Xue Li).

^1^ These authors (Tongxin Jiang and Sijie Li) contributed equally to this work.

**1.** **Design of morphology of photon emission surface**

The two main faces of the GAGG:Ce scintillating waveguides were polished, and no reflective film was applied for avoiding the additional electron absorption. The refractive index of the scintillator is approximately 1.96 at the emission wavelength, resulting in increased reflection at the air interface. The edge faces of the GAGG:Ce scintillating waveguides were ground to form a frosted surface. It was observed that the light output from the frosted surface is higher than that from both the polished surface and the surface with an anti-reflection film. A comparison of normalized surface emissivity of different GAGG:Ce surfaces is presented in Table S1. The surface morphologies of the polished GAGG:Ce surface, which were measured using an atomic force microscope (Cypher S, Asylum Research), are depicted in Figure S1a and S1b. Additionally, the morphologies of the frosted surfaces, which were analyzed using a profile-analyzing laser microscope (VK-X250K, Keyence) and a step profiler (Dektak-XT, Bruker), are illustrated in Figure S1c and S1d.

Table S1 Normalized surface emissivity of different GAGG:Ce surfaces

| **Samples** | **Polished (a.u.)** | **Antireflective (a.u.)** | **Rough(a.u.)** |
| --- | --- | --- | --- |
| 2 mm-thick GAGG:Ce | 1 | 1.104 | 1.386 |
| 1 mm-thick GAGG:Ce | 1 | 1.006 | 1.481 |


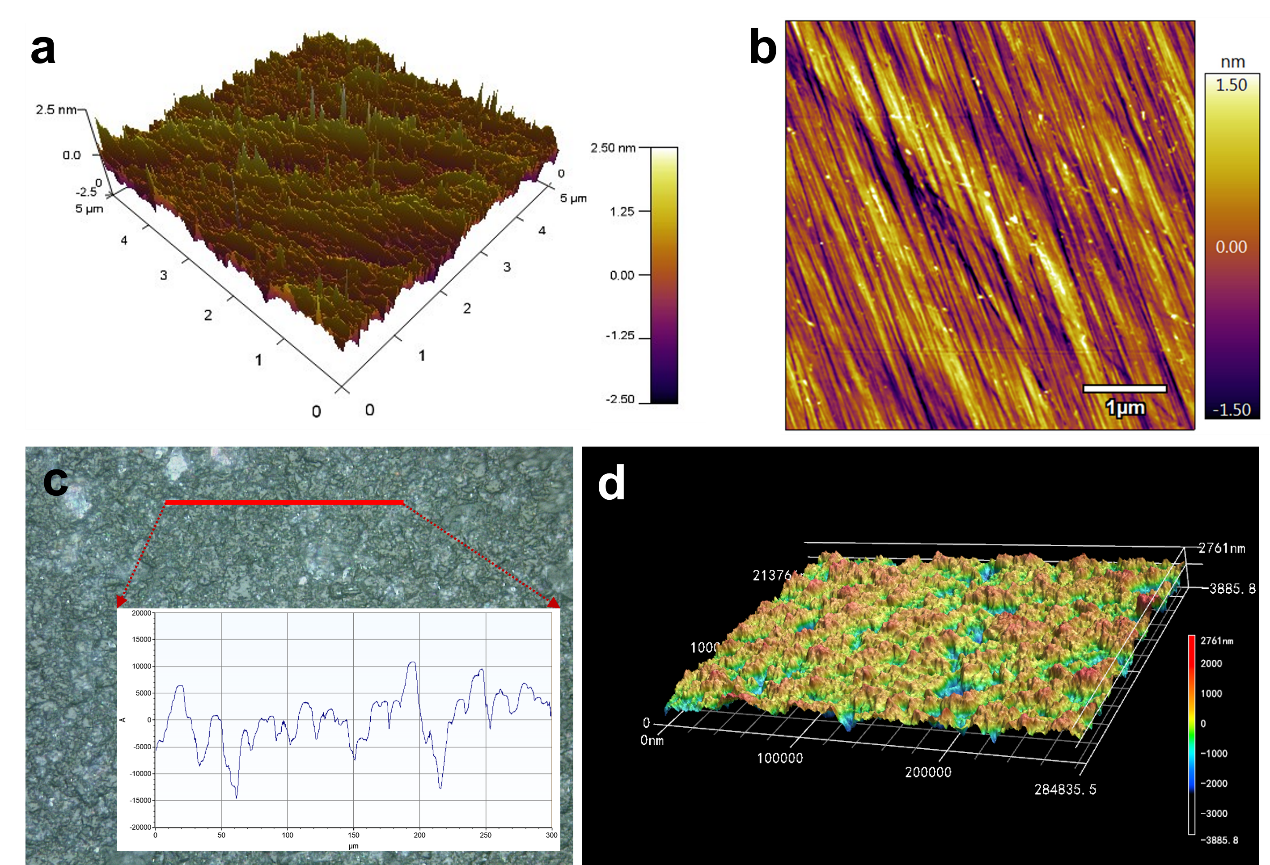


Figure S1. a. Three-dimensional-view and b. top-view morphologies of the polished surface of GAGG:Ce scintillating waveguide. c. Step roughness and d. three-dimensional-view morphology of the frosted surface of GAGG:Ce scintillating waveguide.

**2. Energy deposition** **density (EDD) of** **β-particles in GAGG:Ce under ^85^Kr radiation**


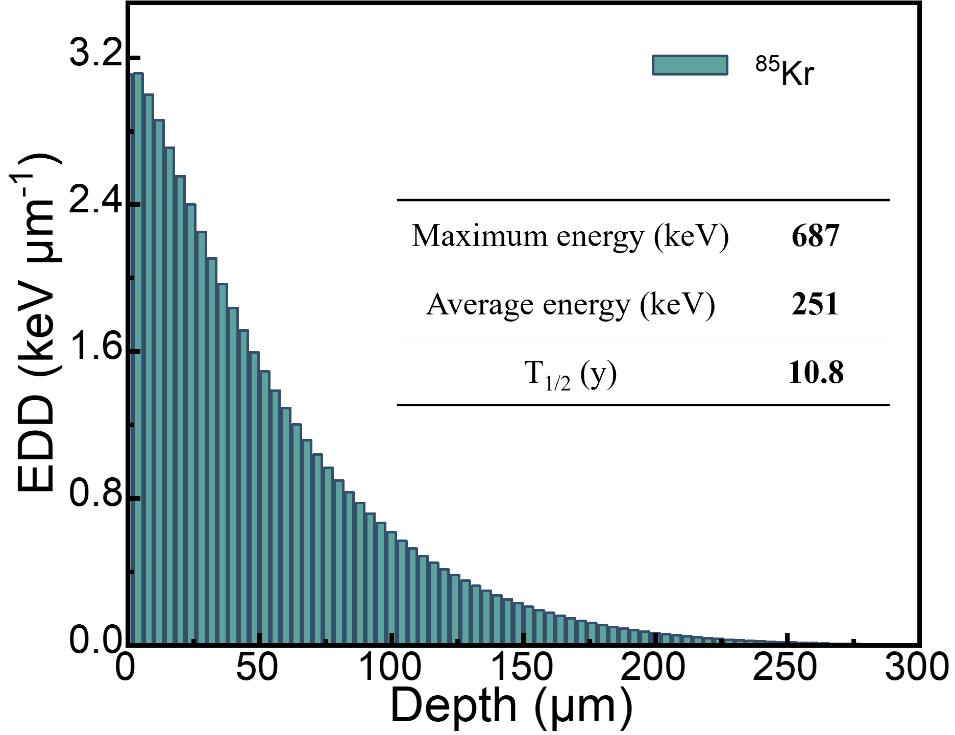


Figure S2. Depth-dependent EDD of β-particles in GAGG:Ce under ^85^Kr radiation.

Table S2 Electron energy spectrum value of ^85^Kr

| Energy  (keV) | Probability  (%) | Energy  (keV) | Probability  (%) | Energy  (keV) | Probability  (%) | Energy  (keV) | Probability  (%) | Energy  (keV) | Probability  (%) |
| --- | --- | --- | --- | --- | --- | --- | --- | --- | --- |
| 17 | 2.847 | 146 | 2.751 | 275 | 2.555 | 405 | 2.029 | 534 | 1.048 |
| 30 | 2.824 | 159 | 2.740 | 288 | 2.522 | 417 | 1.956 | 547 | 0.925 |
| 43 | 2.813 | 172 | 2.729 | 301 | 2.482 | 430 | 1.872 | 560 | 0.808 |
| 56 | 2.802 | 185 | 2.712 | 314 | 2.443 | 443 | 1.782 | 573 | 0.690 |
| 69 | 2.802 | 198 | 2.695 | 327 | 2.393 | 456 | 1.681 | 585 | 0.572 |
| 82 | 2.791 | 211 | 2.673 | 340 | 2.354 | 469 | 1.592 | 598 | 0.466 |
| 95 | 2.779 | 224 | 2.656 | 353 | 2.292 | 482 | 1.485 | 611 | 0.371 |
| 107 | 2.774 | 237 | 2.639 | 366 | 2.236 | 495 | 1.384 | 624 | 0.281 |
| 120 | 2.768 | 250 | 2.611 | 379 | 2.180 | 508 | 1.278 | 637 | 0.191 |
| 133 | 2.757 | 262 | 2.589 | 392 | 2.102 | 521 | 1.160 | 650 | 0.119 |

**3. Electron energy spectra of ^90^Sr and ^90^Sr/Y**


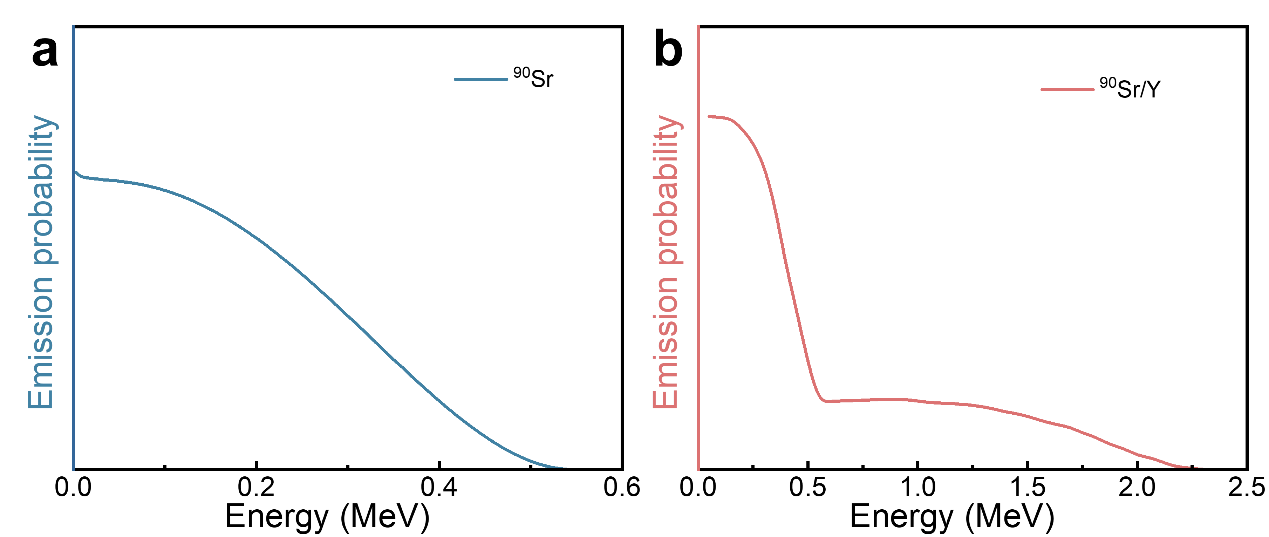


Figure S3 Decay energy spectra of a. ^90^Sr and b. ^90^Sr/Y.

Table S3 Electron energy spectrum value of ^90^Sr

| Energy  (keV) | Probability  (%) | Energy  (keV) | Probability  (%) | Energy  (keV) | Probability  (%) | Energy  (keV) | Probability  (%) | Energy  (keV) | Probability  (%) |
| --- | --- | --- | --- | --- | --- | --- | --- | --- | --- |
| 6 | 3.335 | 106 | 3.140 | 206 | 2.577 | 306 | 1.686 | 406 | 0.726 |
| 16 | 3.298 | 116 | 3.102 | 216 | 2.498 | 316 | 1.587 | 416 | 0.640 |
| 26 | 3.286 | 126 | 3.061 | 226 | 2.417 | 326 | 1.487 | 426 | 0.558 |
| 36 | 3.275 | 136 | 3.014 | 236 | 2.333 | 336 | 1.387 | 436 | 0.479 |
| 46 | 3.266 | 146 | 2.964 | 246 | 2.246 | 346 | 1.287 | 446 | 0.405 |
| 56 | 3.253 | 156 | 2.910 | 256 | 2.157 | 356 | 1.193 | 456 | 0.335 |
| 66 | 3.239 | 166 | 2.851 | 266 | 2.066 | 366 | 1.097 | 466 | 0.271 |
| 76 | 3.220 | 176 | 2.788 | 276 | 1.972 | 376 | 0.999 | 476 | 0.212 |
| 86 | 3.197 | 186 | 2.721 | 286 | 1.878 | 386 | 0.906 | 486 | 0.159 |
| 96 | 3.170 | 196 | 2.650 | 296 | 1.778 | 396 | 0.815 | 496 | 0.113 |

Table S4 Electron energy spectrum value of ^90^Sr/Y

| Energy  (MeV) | Probability  (%) | Energy  (MeV) | Probability  (%) | Energy  (MeV) | Probability  (%) | Energy  (MeV) | Probability  (%) | Energy  (MeV) | Probability  (%) |
| --- | --- | --- | --- | --- | --- | --- | --- | --- | --- |
| 0.05 | 7.965 | 0.55 | 1.523 | 1.05 | 1.511 | 1.55 | 1.124 | 2.05 | 0.275 |
| 0.1 | 7.940 | 0.6 | 1.536 | 1.1 | 1.498 | 1.6 | 1.049 | 2.1 | 0.200 |
| 0.15 | 7.915 | 0.65 | 1.548 | 1.15 | 1.486 | 1.65 | 0.999 | 2.15 | 0.100 |
| 0.2 | 7.690 | 0.7 | 1.553 | 1.2 | 1.473 | 1.7 | 0.936 | 2.2 | 0.050 |
| 0.25 | 7.365 | 0.75 | 1.560 | 1.25 | 1.448 | 1.75 | 0.824 | 2.25 | 0.025 |
| 0.3 | 6.866 | 0.8 | 1.573 | 1.3 | 1.411 | 1.8 | 0.749 | 2.3 | 0.000 |
| 0.35 | 5.942 | 0.85 | 1.585 | 1.35 | 1.361 | 1.85 | 0.624 | 2.35 | 0.000 |
| 0.4 | 4.594 | 0.9 | 1.585 | 1.4 | 1.298 | 1.9 | 0.524 | 2.4 | 0.000 |
| 0.45 | 3.545 | 0.95 | 1.573 | 1.45 | 1.261 | 1.95 | 0.449 | 2.45 | 0.000 |
| 0.5 | 2.397 | 1 | 1.548 | 1.5 | 1.198 | 2 | 0.325 | 2.5 | 0.000 |

**4. Calculation method for energy conversion efficiency in RPVC**

Energy conversion efficiency calculation formula:

$$\eta\text{=}\frac{P_{\max}}{P_{\text{source}}}\text{=}\frac{P_{\max}}{A_{\text{source}}\times3.7\times10^{10}\times E_{\text{source}}\times1000\times1.6\times10^{-19}}\times100\text{\%}$$

here, *η* is the energy conversion efficiency; *P*_max_ is the maximum output power of the RPVC, the unit is W; *P*_source_ is the decay energy from radioisotope source, the unit is W; *A*_source_ is the activity of the radioisotope source loaded in the RPVC, the unit is Ci; *E*_source_ is the average decay energy of radioisotope source, which is 195.8 keV for ^90^Sr.

**5. Summary of materials and performance of RPVCs**

**Table S5** A comparison of reported RPVCs in materials and performance.

| **Radiation source** | **Photovoltaic cell** | **Radioluminescent material** | ***P*_max_**  **(nW)** | **ECE**  **(%)** | **Reference** |
| --- | --- | --- | --- | --- | --- |
| ^3^H | Si | ZnS | 575 | 0.34 | S1 |
| ^63^Ni | Si | ZnS | 0.125 | 0.10 | S2 |
| ^63^Ni | InGaP | ZnS:Cu,Al | 3.35 | 0.29 | S3 |
| ^90^Sr | Si | YAG:Ce | 27.5 | 0.32 | S4 |
| ^238^Pu | GaAs | YPO_4_:Eu | 0.017 | 1.40 | S5 |
| ^3^H | Si | ZnS:Cu | 3630 | 0.60 | S6 |
| ^63^Ni | AlGaInP | ZnS:Cu | 0.62 | 1.33 | S7 |
| ^90^Sr | GaAs | LYSO:Ce | 610 | 0.92 | S8 |
| ^243^Am | Cs_0.05_MA_0.1_FA_0.85_PbI_3_ | TbMel | 1.538 | 0.89 | S9 |
| **^90^Sr** | **AlGaInP** | **GAGG:Ce** | **48900** | **2.96** | **This work** |

**Reference**

S1 Walton, R. et al. Radioisotopic battery and capacitor system for powering Wireless Sensor Networks. *Sensors and Actuators A-Physical* **203**, 405-412, [doi: 10.1016/j.sna.2013.09.010](https://doi.org/10.1016/j.sna.2013.09.010) (2013).

S2 Xu, Z. H. et al. Development of a beta radioluminescence nuclear battery. *Nuclear Science and Techniques* **25**, 4, doi: 10.13538/j.1001-8042/nst.25.040603 (2014).

S3 Russo, J. et al. A radioluminescent nuclear battery using volumetric configuration: ^63^Ni solution/ZnS: Cu, Al/InGaP. *Applied Radiation and Isotopes* **130**, 66-74, doi: 10.1016/j.apradiso.2017.09.018 (2017).

S4 Lei, Y. S. et al. Demonstration and aging test of a radiation resistant strontium-90 betavoltaic mechanism. *Applied Physics Letters* **116**, 153901, doi: 10.1063/1.5140780 (2020).

S5 Prudchenko, K. K. et al. Investigation of the characteristics of a radioisotope source based on a (Y)PO_4_/(^238^Pu) self-glowing crystal and an Al_x_Ga_1-x_As/GaAs photovoltaic converter. *Journal of Physics: Conference Series* **2103**, 012195, doi: 10.1088/1742-6596/2103/1/012195 (2021).

S6 Phatangare, A. B. et al. Novel nuclear batteries based on radioluminescence. *Energy Technology* **10**, 2200285, doi: 10.1002/ente.202200285 (2022).

S7 Jiang, T. X. et al. ^63^Ni-based radioluminescent isotope cells with enhanced photon transport interfaces. *Journal of Science*: *Advanced Materials and Devices* **8**, 100611, doi: 10.1016/j.jsamd.2023.100611 (2023).

S8 Cui, Q. M. et al. A ^90^Sr/^90^Y-radioisotope battery based on betavoltaic and beta-photovoltaic dual effects. *Materials Science in Semiconductor Processing* **179**, 108493, doi: 10.1016/j.mssp.2024.108493 (2024).

S9 Li, K. et al. Micronuclear battery based on a coalescent energy transducer. *Nature* **633**, 811-815, doi: 10.1038/s41586-024-07933-9 (2024).
